# Supplementary material for: Trait and plasticity evolution under competition and mutualism in evolving pairwise yeast communities
Source: PLoS One. 2025 Jan 15;20(1):e0311674. doi: 10.1371/journal.pone.0311674 (PMC11734945; doi:10.1371/journal.pone.0311674)

Supporting Information Figure 1. Ancestral values of growth rate and yield at 24 hrs. for strains used in experimental evolution experiments. Strains deficient in adenine production (CLs or MLs) had significantly reduced growth rates ($F_{3,188}$=17.84, P <0.0001), but higher population sizes at 24 hrs ($F_{3,188}$=660.67, P < 0.0001, P <0.0001). Error bars are standard errors. Grey colored bars are strains that produce lysine at wildtype (light grey) or overproduce lysine (dark grey) and green colored bars are strains that produce adenine at wildtype levels (light green) or overproduce adenine (dark green).

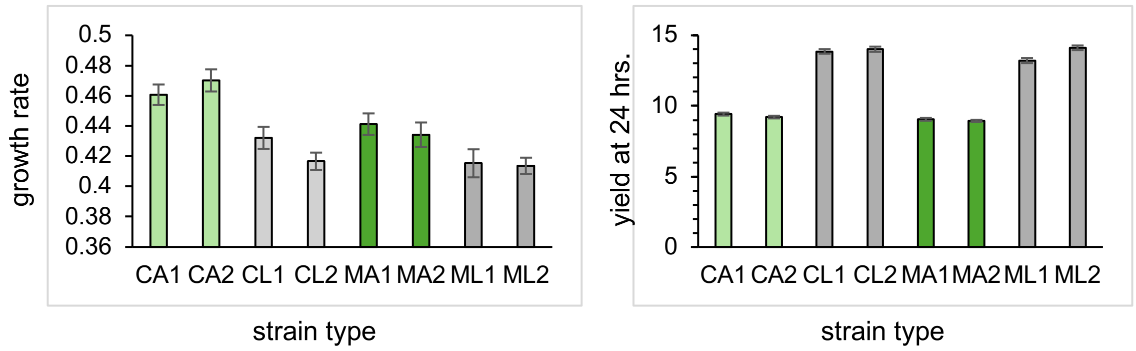

Supplement: S1 Fig — Strains deficient in adenine production (CLs or MLs) had significantly reduced growth rates (F3,188 = 17.84, P <0.0001), but higher population sizes at 24 hrs (F3,188 = 660.67, P < 0.0001, P <0.0001). Error bars are standard errors. Grey colored bars are strains that produce lysine at wildtype (light grey) or overproduce lysine (dark grey) and green colored bars are strains that produce adenine at wildtype levels (light green) or overproduce adenine (dark green). (PDF) [file pone.0311674.s001.pdf]
