# Supplementary material for: Trait and plasticity evolution under competition and mutualism in evolving pairwise yeast communities
Source: PLoS One. 2025 Jan 15;20(1):e0311674. doi: 10.1371/journal.pone.0311674 (PMC11734945; doi:10.1371/journal.pone.0311674)
Supplement: S1 Table — (DOCX) [file pone.0311674.s002.docx]

**Table S1. Strain identification and genotypes for strains used in experimental evolution experiments**.

| **Strain ID** | **Genotype** | **Label** |
| --- | --- | --- |
| RY1045 | MATa ste3ΔkanMX4 ade8Δ0 leu2Δ0 ura3Δ0 LYS21OP | ML1 |
| RY1048 | MATa ste3ΔkanMX4 ade8Δ0 leu2Δ0 ura3Δ0 LYS21WT | CL1 |
| RY1051 | MATa ste3ΔkanMX4 ade8Δ0 his3Δ1 ura3Δ0 LYS21OP | ML2 |
| RY1054 | MATa ste3ΔkanMX4 ade8Δ0 his3Δ1 ura3Δ0 LYS21WT | CL2 |
| RY1063 | MATa ste3ΔkanMX4 lys2Δ0 his3Δ1 ura3Δ0 ADE4OP | MA1 |
| RY1066 | MATa ste3ΔkanMX4 lys2Δ0 his3Δ1 ura3Δ0 ADE4WT | CA1 |
| RY1069 | MATa ste3ΔkanMX4 lys2Δ0 leu2Δ0 ura3Δ0 ADE4OP | MA2 |
| RY1072 | MATa ste3ΔkanMX4 lys2Δ0 leu2Δ0 ura3Δ0 ADE4WT | CA2 |
