# Supplementary material for: Trait and plasticity evolution under competition and mutualism in evolving pairwise yeast communities
Source: PLoS One. 2025 Jan 15;20(1):e0311674. doi: 10.1371/journal.pone.0311674 (PMC11734945; doi:10.1371/journal.pone.0311674)
Supplement: S2 Table — Strains with different genetic markers were used to facilitate separation on selection plates. (DOCX) [file pone.0311674.s003.docx]

**S2 Table. Experimental communities and strain pairings used in experimental evolution experiments.** Strains with different genetic markers were used to facilitate separation on selection plates.

| **Strain type** | **Community type** | | |
| --- | --- | --- | --- |
|  | **single** | **mutualism** | **competition** |
| 1063 **^_^**his (MA) | x | a |  |
| 1069 **^_^**leu (MA) | x | b |  |
| 1045 **^_^**leu (ML) | x | a |  |
| 1051 **^_^**his (ML) | x | b |  |
| 1048 **^_^**leu (CL) | x |  | c |
| 1054 **^_^**his (CL) | x |  | d |
| 1066 **^_^**his (CA) | x |  | c |
| 1072 **^_^**leu (CA) | x |  | d |
