# Supplementary material for: Trait and plasticity evolution under competition and mutualism in evolving pairwise yeast communities
Source: PLoS One. 2025 Jan 15;20(1):e0311674. doi: 10.1371/journal.pone.0311674 (PMC11734945; doi:10.1371/journal.pone.0311674)
Supplement: Attachment — Submitted filename: PLOSONEreviews_Response.docx [file pone.0311674.s004.docx]

Wang et al., “Trait and plasticity evolution under competition and mutualism in evolving pairwise yeast communities”

Reviewer's Responses to Questions

**Comments to the Author**

5. Review Comments to the Author

Please use the space provided to explain your answers to the questions above. You may also include additional comments for the author, including concerns about dual publication, research ethics, or publication ethics. (Please upload your review as an attachment if it exceeds 20,000 characters)

**Reviewer #1:** This manuscript presents an experimental investigation into the evolutionary responses of genetically modified brewer's yeast to competition and mutualism. The study highlights the impact of biotic interactions, such as competition and mutualism, on growth rate and resource utilization efficiency (RUE) phenotypes. The authors draw the conclusion that the existence of competitive partners decreased RUE and established a significant tradeoff between growth rate and RUE. Conversely, having mutualistic partners resulted in marginally lower growth rates but increased RUE. Furthermore, the authors found that biotic interactions had no significant effect on either growth rate plasticity or RUE plasticity.

This study provides valuable insights into the effects of biotic interactions on trait and plasticity evolution, which is an area that has received limited attention. The Introduction and Discussion sections are well-crafted, effectively summarizing the main points and clearly outlining the study's objectives and findings. However, the Methods and Results sections could benefit from further refinement, particularly with regard to the description of the growth measurements. Moreover, the presented study results and figures are currently insufficient to demonstrate the validity of the reported outcomes convincingly. Therefore, the authors need to revise their manuscript in accordance with the following feedback and recommendations.


\## Major comments

1. The growth measurement experiments are poorly described, which poses a significant challenge in comprehending and interpreting the outcomes

*We added additional detail about the growth experiments including which strains were measured at certain time points and why (Lines 249-254*). *The methodology in terms of type of media, time for growth, and growth rate calculations are already provided.*

a. The Results section does not include a summary of the evolution and growth measurement experiments, making it difficult to interpret the figures without a thorough review of the Methods section.

*We were not clear about what the reviewer meant by a summary so we added the following to help remind the reader about the general design and goals of each experiment/measurement in the Results section.*

*Line 345-347 “To provide a baseline for trait evolution, ancestral strains were grown individually in both media types and measured for growth rate at 4, 6 and 24 hrs. via optical density. Growth rate was determined by the difference in growth between 4 and 6 hrs.”*

*Lines 358-359 “Strains were grown alone to understand how the culturing environment affected trait evolution, and these evolved strains were used as the control with which to compare the effects of competition and mutualism.”*

b. Figure 1A presents some ambiguity regarding the competition between the strains, as only Adenine and Lysine are depicted as being taken up. Additionally, the color codes utilized in the figure lack a clear explanation, further contributing to potential confusion. To ensure a comprehensive understanding of the presented data, it is advisable for the authors to provide a more detailed schematic and a more thorough description.

*We revised the figure to indicate what strains were competing for and added the following to the figure legend, “Grey colors denote strains that are produce adenine at normal, wild type levels or overproduce adenine but are deficient in producing lysine, and green colors denote strains that produce lysine at normal, wild type levels or overproduce lysine but are deficient in producing adenine.”*

c. The methodology regarding the growth measurements is somewhat ambiguous. The authors have not specified the number of strains for which growth rates and yields were measured, nor have they provided information regarding which strains were grouped together. Phrases like "When possible, we assayed similar mutualist and competitor strains at the same time" (L248) have left room for interpretation. Given that the description of these experiments is crucial to the study, it would benefit from further refinement.

*Agreed. We added a supplemental table with the strain marker information, strain number, and the abbreviation used in the manuscript. We also added another supplemental table that identified the pairings used in the evolution experiments.*

*We also added the following text to the methods (Lines249-254). “We grouped strains by their initial strain type, (MA: Ade^OP^Lys^-^, ML: Ade^-^Lys^OP^, CA: Ade^WT^Lys^-^, or CL: Ade^-^Lys^WT^ ) for these growth assays so that, measurements of ancestral and evolved strains from each treatment were done together and directly comparable (Table S2). Each group of strains contained one evolved strain from all possible treatments and two colonies of the ancestral strain. For example, an ancestral MA strain and all of its evolved MA descendants were measured at the same time.*

2. Regarding line 259, it was specified that the yield was computed at the end of the 24-hour interval, as this was when the highest population size was attained throughout the evolutionary experiment. It is critical for the authors to confirm that the low-resource environment is subjected to identical circumstances or, at the very least, ensure that the cells in the culture have not been starved of nutrients for an extended period. Since, the deficiency of adenine and lysine can result in cell death, which may affect OD measurements.

*We added a reference (Vidal et al 2020) that examined this potential issue and found that almost all of the cultures contain live cells and death due to starvation does not start until after 24 hrs. Lines(264-266)*

3. According to the authors, the trait and plasticity responses in the LMMs seem to have been minimally affected by strain types. However, it is vital to consider that genetic differences alone could result in varying growth rates among the ancestor strains, which could impact the culture's temporal resource dynamics in various ways (Vidal et al., 2020).
a. The authors did not evaluate how pairing a strain with a different mutant might impact the mutualistic and competitive treatments.

*This is an interesting point but well beyond the scope of our study. Our choice of strain pairings was planned so that the same marker types were used to account for differences in traits due to marker types. One could argue that each replicate for each pair had the opportunity to evolve in unique ways during the course of the experiment that would allow for the evolution of mutants. There will probably be effects due to using different mutants at the starting point, but we argue that based on the lack of differences among strains (even those that overproduced adenine and lysine) in trait evolution within each treatment suggests that selection was strong and the results are robust.*

b. Currently, the LMM model does not consider the traits of the partner strain and how it affects the evolution of traits and plasticity. Can the authors provide any insights into this?

*This is another interesting idea, but was beyond the scope of this study. Ancestral strains did differ in growth rate and yield (see Supplemental Fig 1), but this was due to whether they were overproducing or had wildtype production of adenine versus lysine. Within each adenine or lysine “guild” there were no differences in initial trait values. We wanted similar trait values initially as a control that would more likely isolate the signature of the biotic interactions rather than initial trait differences.*

4. The source of the data used to produce Figure 3 is unclear, and there is no definitive indication provided in the caption, results, or methods sections as to how the calculations were performed and the values presented were interpreted. Whether these scores represent differences in trait and plasticity values from the growth experiments remains unclear. It is recommended that the authors supplement the results section with a brief explanation to address this issue.

*We have rearranged text and added additional text to the methods to explain what data were used for these analyses. Lines 321-327. “We first tested whether the genetic differences among the experimental strains were important by using models containing the strain type (either Ade^-^ or Lys^-^) and the marker genotype (His^-^ or Leu^-^) and their interaction as covariates. These genetic effects were minimal for all models, so we simplified all the models to have only strain identity as a random effect to simplify model interpretation. Given this result, we then pooled data on trait values from strains used in each of the evolutionary treatments. In the comparison for trait values between ancestral strains and those evolved alone, we calculated average traits values using all strain types, (MAs, MLs, CAs, CLs). For comparisons between strains evolved alone or with a competitor we pooled data from the CA and CL strains, and between strains evolved alone or with a mutualist we pooled data from the MA and ML strains.”*

*We also added the following text to the figure legend.”* *Data for each biotic interaction were pooled across all strains involved (i.e, all mutualist strains, all competitor strains).”*

a. The authors may need to reconsider their results as the average effects in most bars are less than the standard errors. What is the source of these errors?

*The large sources of error in Fig 3 (now Fig 4) are result of strains in each replicate pair evolving along independent evolutionary trajectories. In some ways, we were excited by this result because it indicates that evolution may send each community in different directions with respect to trait evolution. This can be seen in Fig 2. The SE for strains evolved alone is quite higher than the ancestral starting point. This is important because it suggests that detecting a signature due to biotic interactions may be difficult without good replication, and in order for those interactions to have a large impact, selection imposed by competition or mutualism would have to be large.*

b. Can the authors clarify in the discussion section whether these effects are large enough to affect the outcomes of dynamics in a community?

*Lines 481-483. We added “Additionally, there was more variance in traits values from strains evolved alone when compared to ancestrals, indicative of the fact that each replicate was free to evolve along a different trajectory (Fig. 2 a,b). This result suggests that evolution in response to an interacting partner would have to be strong enough to overcome this additional noise added by replicate communities evolving independently from one another.”*

5. The authors should present a plot with the actual trait and plasticity values for all strains and treatments, either as a main or supplemental figure. This will provide information on the distribution of traits and plasticity values due to different mutations.

*We have included a new figure (Fig. 2) with the trait data requested.*

6. Figure 4 should be supplemented with a calculation of the Spearman correlation coefficient between the Δgrowth rate and the ΔRUE due to the potential non-linear relationship between the two variables. This addition will provide a more comprehensive understanding of the relationship between Δgrowth rate and ΔRUE and help to identify any strong correlations that may exist.

*The correlation correlations are now included in the figure legend after the regression coefficients.*

\## Minor comments

7. (Line 351) Fig. 2C does not show growth rate plasticity.

*This sentence is now deleted.*

8. Line 353's statement, "little difference in growth plasticity," contradicts Line 350's statement, "Growth plasticity was slightly higher."

*Agreed. We deleted the sentence “Growth plasticity was slightly greater for strains evolved with a competitive partner, although this effect was only marginally significant (Table 1, Fig. 3c, Fig. 4c)” because this result was not significant.*

9. Though Figure 2C shows lower growth rates for partner strains due to mutualistic interactions compared to strains evolved alone, Table 1 (and Figure 3A) indicates no significant difference. Can the authors explain why this is the case?

*Yes. The issue here is that the reaction norm for strains evolving alone contains both competitive and mutualist strains and was not the reaction norm used for testing for the effects of competition or mutualism. The evolving alone reaction norm was based on just competitor or mutualist strains in the statistical analyses. We removed the alone line from panels c and d as we agree with the reviewer that this is confusing and also not accurate. We added the following to the figure legend to be more explicit. “Data from all strains in each treatment and community context were pooled together as there were no differences among strains within each treatment. Statistical comparisons of trait evolution between evolving with a competitor or a mutualist versus alone are reported Table 1.”*

10. In line 408, the authors refer to Lin et al., 2020 while stating that "a reduction in efficiency is a common response of microbes to serial transfer culturing methods." However, Lin et al., 2020 show that serial dilutions affect growth rates but not yields (RUE). Could the authors clarify this contradiction?

*The reviewer is correct about the Lin et al 2020 paper. That paper is a modeling paper. We have removed it from this citation list for the sentence. The Jasmin et al 2102 paper was an empirical paper on yeast that demonstrated the pattern we were citing and we added Bachmann et al. 2016 which is a review of culturing methods and their effects on growth rate and RUE in microbes.*

**Reviewer #2:** In this manuscript, Wang et al. have experimentally tested how competition and mutualism affects trait and phenotypic plasticity in pairwise communities of genetically modified brewer’s yeast. They have constructed competitive and mutualistic communities, by combining yeast strains with different mutations in key metabolic genes. They have quantified and compared the evolutionary changes in growth rate, resource use efficiency, and their plasticity in strains evolving alone, with a competitor, and with a mutualist. Their experiments have revealed that strains evolving with a competitive partner had higher growth rates, slightly lower efficiency, and a stronger tradeoff between growth rate and efficiency. In contrast, their results indicate that mutualistic strains had slightly lower growth rates, higher efficiency, and a weak evolutionary tradeoff between growth rate and efficiency. Furthermore, they have observed that competition and mutualism had little effect on plasticity evolution for either trait.

The experiments are well-designed for testing the main hypotheses. The results are significant, and the data supports the main conclusions. However, I have the following minor comments:

1. In lines (172-175), it is mentioned that “Vidal et al (2020) demonstrated that lysine deficient strains die at a faster rate than adenine deficient strains, when starved for the corresponding essential nutrient. This asymmetry in nutrient function may influence evolutionary changes in resource use, growth and population dynamics for different strain types.”.
It is not clear for me how the authors ensure that this asymmetry in nutrient function does not influence their comparisons. Especially, it is mentioned in lines (220-222) that the size of the communities, which are compared in this study is not equal (75 single, 56 mutualistic and 35 competitive communities), because some communities have gone extinct during the 222 experiment. Thus, my main concern here is that the fraction of adenine-deficient-stains vs. lysine-deficient-strains might not stay the same in these communities, and hence the mentioned asymmetry in nutrient function might have acted as a major confounding factor influencing their conclusions. I strongly recommend the authors to clarify and discuss this point.

*There is no question that the population sizes of the different strain types will fluctuate throughout the experiment. First, the serial dilution process will influence ratios during each transfer event. Communities that went extinct suggests that indeed changes in strain population sizes was happening and that it was likely a compounding effect—smaller starting population means smaller population at the next transfer event, etc. until eventual extinction. Second inherent growth rate differences among strains will result in different population sizes of each strain in a paired culture. Even so, these effects are controlled across interaction type because we used similar null genotypes for the competitor strains and the mutualistic strains.*

*Lines 225-299. We added the following to the section on growth experiments to indicate this. “Extinction of paired strain communities was due to a combination of differences in growth rates among Ade^-^ and Lys^-^ strains, the serial dilution process itself, and interaction type. The use of similar null alleles for the competitor strains and mutualist strains served as a control for initial strain differences.”*

2. I believe that following sentence in lines (86-88) “Thus, disentangling the importance of biotic interactions from abiotic factors is necessary for understanding how phenotypic plasticity may evolve in general for interacting species” is not well-connected to the preceding sentences, and I think a logical connection is missing here, which might cause confusion. The preceding sentences are focused on competition vs. mutualism, while the final sentence (86-88) suddenly shift the focus towards biotic vs. abiotic interactions, and thus I recommend the authors to rewrite this sentence.

*Agreed. We removed the ambiguity of this sentence be changing it to “Thus, disentangling the importance of biotic interactions such as competition and mutualism from abiotic factors is necessary for understanding how phenotypic plasticity may evolve in interacting species (Scheiner et al. 2015).*”

3. It seems one of the abbreviations for the strain names in line 172 is incorrect:
“lysine deficient strains (AdeOP Lys- and Ade- LysWT)”
Ade- LysWT is not a lysine-deficient strain.

*Correct. Now changed to “…that lysine deficient strains (Ade^OP^Lys^-^ and Ade^WT^Lys^-^)”*
