# Supplementary material for: Trait and plasticity evolution under competition and mutualism in evolving pairwise yeast communities
Source: PLoS One. 2025 Jan 15;20(1):e0311674. doi: 10.1371/journal.pone.0311674 (PMC11734945; doi:10.1371/journal.pone.0311674)
Supplement: Attachment — Submitted filename: ReviewersCommentsRevision_R2.docx [file pone.0311674.s005.docx]

**Response to Reviewers’ comments**

Reviewer #1: The manuscript presents a valuable study illustrating how biotic interactions, including both competition and mutualism, influence the growth rate and resource utilization efficiency (RUE) phenotypes within a microbial community. The authors have modified the manuscript in response to the feedback and suggestions provided by the reviewers, effectively addressing most of the reviewers’ concerns. The revisions incorporated into the Results section, along with the added supplementary material, have notably enhanced the clarity and comprehensiveness of the study. Below are a few final comments:

\## Major comments
In Line 225 the authors mention that the “Extinction of paired strain communities was due to a combination of differences in growth rates among Ade- and Lys- strains, the serial dilution process itself, and interaction type”. However, based on Figure 3, the growth rate and RUE of the CA2 strain are similar to those of CA1, and likewise, the values for CL1 are comparable to CL2. In light of this, it would be beneficial for the authors to offer an explanation regarding the potential factors that led to the extinction of the CA2 strain.

Without doing additional sets of experiments we cannot be sure of the causes, but we added the following to the Results section lines (227-234) to discuss the possibilities.

*“Extinction of paired strain communities was likely due to a combination of slight differences in growth rates among Ade^-^ and Lys^-^ strains, the serial dilution process itself, and interaction type. For example, the serial dilution process produces a population bottleneck during the transfer event. Any factor such as competition, reduced population size of mutualist partner or sampling error that reduces the size of the transferred populations will start a downward trend in a strain’s population size. This process gets compounded during the serial dilution process as only 10% of the total community is transferred. The fact that single strain communities never went extinct suggests that interacting with a partner strain places additional selective pressure on strains that will influence long term persistence.”*

\## Minor comments
1) Figure 1: the authors could consider including an annotation for Figure 1a, analogous to the annotation present in Figure 1b, such as: “media contains Ade and Lys, and the strains compete for all other nutrients”

Agreed and we have amended the figure as requested.

2) Figure 3: The quality of the image is poor and appears blurry

3) (Line 343) “growth rate at 4, 6 and 24 hours”. It might be more suitable to use 'yield' instead of 'growth rate'.

*“We changed this to “To provide a baseline for trait evolution, ancestral strains were grown individually in both media types and measured for population size at 4, 6 and 24 hrs. via optical density.”*

4) (Line 347-348) Is this referring to Figures 2a and 2b instead of Figures 3a and 3b? Figure 3 does not compare low and high resource environments.

*Corrected*

5) (Line 359-361) Figure 2 does not show growth rate plasticity or RUE plasticity

*Corrected*

6) (Line 368) Change Figure 4a to Figure 4, since 4a only displays the growth rate

*Corrected*
